# Unified Model for Laser Doping of Silicon from Precursors

**DOI:** 10.3390/ma14092322

**Published:** 2021-04-29

**Authors:** Mohamed Hassan, Morris Dahlinger, Jürgen R. Köhler, Renate Zapf-Gottwick, Jürgen H. Werner

**Affiliations:** Institut für Photovoltaik (ipv), University of Stuttgart, Pfaffenwaldring 47, 70569 Stuttgart, Germany; dahlinger.ipv@gmx.de (M.D.); juergen.koehler@ipv.uni-stuttgart.de (J.R.K.); renate.zapf-gottwick@ipv.uni-stuttgart.de (R.Z.-G.); Juergen.Werner@ipv.uni-stuttgart.de (J.H.W.)

**Keywords:** laser processing, laser doping, boron oxide properties, emitter, silicon solar cell, IBC, SIMS

## Abstract

Laser doping of silicon with the help of precursors is well established in photovoltaics. Upon illumination with the constant or pulsed laser beam, the silicon melts and doping atoms from the doping precursor diffuse into the melted silicon. With the proper laser parameters, after resolidification, the silicon is doped without any lattice defects. Depending on laser energy and on the kind of precursor, the precursor either melts or evaporates during the laser process. For high enough laser energies, even parts of the silicon’s surface evaporate. Here, we present a unified model and simulation program, which considers all these cases. We exemplify our model with experiments and simulations of laser doping from a boron oxide precursor layer. In contrast to previous models, we are able to predict not only the width and depth of the patterns on the deformed silicon surface but also the doping profiles over a wide range of laser energies. In addition, we also show that the diffusion of the boron atoms in the molten Si is boosted by a thermally induced convection in the silicon melt: the Gaussian intensity distribution of the laser beam increases the temperature-gradient-induced surface tension gradient, causing the molten Si to circulate by Marangoni convection. Laser pulse energy densities above *H* > 2.8 J/cm^2^ lead not only to evaporation of the precursor, but also to a partial evaporation of the molten silicon. Without considering the evaporation of Si, it is not possible to correctly predict the doping profiles for high laser energies. About 50% of the evaporated materials recondense and resolidify on the wafer surface. The recondensed material from each laser pulse forms a dopant source for the subsequent laser pulses.

## 1. Introduction

Edge isolation [1], texturing [2], ablation [3,4], cutting [5], and annealing of implanted silicon [6,7] are some of the common applications of laser processing for solar cell fabrication. Laser doping of silicon with a precursor layer that serves as a dopant source [4,8,9,10] shows significant advantages compared with conventional furnace diffusion processes. Laser processing enables the selective doping of silicon with high spatial resolution.

Laser doping of silicon relies on melting the silicon’s surface with a short laser pulse, which melts the silicon. Then, doping atoms (such as boron or phosphorus) contained in a thin precursor layer diffuse into the silicon melt. After the (defect-free) recrystallization of the Si melt, the doping atoms are contained in the solid Si. The detailed tailoring of the doping profiles requires not only a qualitative but also a quantitative understanding of the physics and chemistry of laser doping. Only under these conditions is it possible to tailor and improve the electrical and electronic properties of the doped surface layers.

In principle, there are three possibilities for the doping from the precursor layer into the silicon.

(i) Solid and Melt/Melt-Doping: Doping of the Si melt from the solid precursor; in this case, the doping precursor does not evaporate but is solved in the Si melt. The Si itself does not evaporate. For example, the doping of Si with the help of a pure, sputtered boron containing the precursor falls in this range. Boron melts at temperature $T_{\mathrm{melt},\;B_{2}O_{3}}$ = 2573 K [11] and evaporates at $T_{\mathrm{evap},\;B_{2}O_{3}}$ = 4203 K [12]; the evaporation of the precursor layer is not relevant for the doping process. Lill et al. [13] reported on this case. They used a boron precursor, a pulsed laser of 532 nm wavelength and a 42 ns pulse duration (at full width at half maximum (FWHM) and medium laser pulse energy densities. For these experimental conditions, their model [13] explained the measured doping profiles well. Unfortunately, the model is not valid if either the precursor or the Si evaporate.

(ii) Gas/Melt-Doping: Doping of the Si melt from the gas phase; in this case, the precursor evaporates and doping of the Si melt takes place from the gas phase. This process resembles classic doping using a doping furnace and a gaseous doping source. In this case, the Si itself also does not evaporate. This kind of doping takes place if one uses either a pure, sputtered phosphorus layer [14] or a spin-on phosphoric acid solution [15]. Phosphorus evaporates at 550 K [12] and crystalline silicon melts at temperature $T_{\mathrm{melt},\;\mathrm{Si}}$ = 1687 K [16]. Köhler et al. [14] also used a pulsed green laser of 532 nm wavelength (7 ns FWHM pulse duration). In their publication [14], they used a model that resembled the description of classic furnace diffusion from a phosphorus silicate layer; their model predicted the experimental diffusion profiles well. However, it is only applicable to laser energy densities below the evaporation threshold of Si. In a similar way, the model of Blecher et al. [15] for experiments using a continuous-wave (CW) laser with 532 nm wavelength is also not applicable to pulsed lasers with a pulse duration in the nanosecond regime. In this case, however, the doping profiles were well predicted for different scanning speeds.

(iii) Gas/Gas-Melt-Doping: Doping of the Si melt from a mixture of evaporated doping source AND evaporated Si. Thus, in this case, parts of the silicon’s surface evaporate too. This kind of doping takes place in the case of medium to high laser energy densities. In the following, we show that this range starts already at about 2.8 J/cm^2^.

The present work presents a unified model, which is able to cover all three possibilities (solid/melt, gas/melt, gas/gas-melt) of laser doping, including the high energy regime in which the doping precursor as well as parts of the silicon’s surface evaporate. We developed a Matlab code for solving the coupled heat and diffusion equations. The present model enables us to predict the melt width $W_{\mathrm{melt}}$ and depth $d_{\mathrm{melt}}$ of the silicon’s surface. Furthermore, it allow us to predict the diffusion profiles over a wide range of laser pulse energy densities *H*.

## 2. Pulse Energy Density Regimes

**Figure 1:**
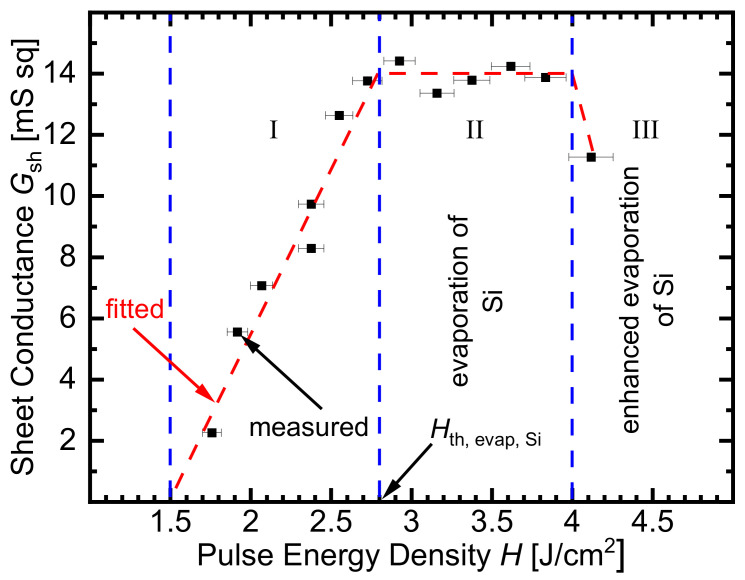
The measured sheet conductance $G_{\mathrm{sh}}$ of the laser-doped silicon surface shows three pulse energy density *H* regimes, when doped with boron. In this case, the precursor consists of sputtered, 1 nm-thick B${}_{2}$O${}_{3}$, covered with 12 nm-thick sputtered, intrinsic a-Si. In the technologically most important regime II, the physics of the doping process is influenced by the evaporation of the silicon’s surface, as shown below. Regime II starts when the used *H* exceeds the threshold energy density H_th. evap, Si_ of silicon evaporation. In regime I (1.5 J/cm${}^{2}$ ≤H≤ 2.8 J/cm${}^{2}$), the sheet conductance $G_{\mathrm{sh}}$ increases linearly because the melting depth of Si, and therefore the total amount of incorporated boron atoms, increases also linearly. In regime III, for *H* > 4 J/cm${}^{2}$, strong evaporation of Si results in a decrease in the conductance $G_{\mathrm{sh}}$ again. Quantitative understanding of the evaporation of precursor and Si during laser doping is therefore a key to modeling, in particular, for the technologically most interesting regime II.

Figure 1 shows three regimes for the measured sheet conductance $G_{\mathrm{sh}}$ of a laser-doped silicon surface. In this case, we dope the silicon from a sputtered boron oxide precursor. Details of the laser system will be discussed below.

To measure the sheet conductance $G_{\mathrm{sh}}$ of the doped silicon surface, an n-type 6-inch Czochralski-grown Si Wafer having a base resistivity $\rho_{\mathrm{base}}$ = 1.5 Ωcm and thickness $d_{\mathrm{wafer}}$ ≈ 158 µm is provided with a sputtered boron oxide (B_2_O_3_) layer of 1 nm thickness, covered with 12 nm sputtered amorphous silicon (a-Si) layer. The surface is then irradiated with a pulsed laser and the sheet resistance $R_{\mathrm{sh}}$ is measured. The sheet conductance depends on the sheet resistance through

(1) $$G_{\mathrm{sh}}\;=\;\frac{1}{R_{\mathrm{sh}}}.$$

For *H* < 1.5 J/cm^2^, no sheet conductance $G_{\mathrm{sh}}$ is measurable. The laser energies are below the melting thresholds of Si; therefore, doping is not possible. In regime I (1.5 J/cm^2^ ≤H≤ 2.8 J/cm^2^), the sheet conductance $G_{\mathrm{sh}}$ of the doped silicon increases linearly, which indicates that the amount of incorporated boron atoms in the doped layer also increases linearly. For 2.8 J/cm^2^ <H≤ 4.0 J/cm^2^, in regime II, the sheet conductance $G_{\mathrm{sh}}$ saturates. This regime is the most important one for technological applications. We will show below that in this regime, parts of the doped silicon surface evaporate. In regime III (*H* > 4 J/cm^2^), the sheet conductance $G_{\mathrm{sh}}$ strongly decreases again. The reason for the behavior of $G_{\mathrm{sh}}$ for *H* > 4 J/cm^2^ lies in the enhanced evaporation of the doped silicon.

## 3. Laser System

### 3.1. Laser Beam

**Figure 2:**
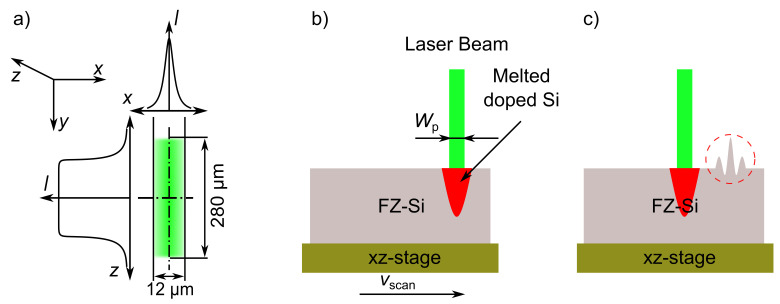
(**a**) Top view of the used laser beam with the line-focused shape with area $A_{\mathrm{spot}}$ of 12 × 280 µm${}^{2}$. The laser beam has a Gaussian intensity *I* distribution in x-direction and a tophat intensity *I* distribution in z-direction. (**b**) The wafer to be irradiated is placed on an xz-translation stage. The translation stage moves in x-direction with a scanning speed $v_{\mathrm{scan}}$ = 300 mm/s; the laser irradiates the sample with a pulse repetition rate *f* = 12.5 kHz. Each laser pulse locally melts the silicon surface. Such a high scanning speed $v_{\mathrm{scan}}$ ensures no overlap of the irradiated and melted areas; therefore, the individually treated spots are clearly separated. (**c**) The melted surface resolidifies after the pulse ends, leaving behind a deformed surface, as discussed later.

Figure 2a describes the shape and intensity *I* distribution of the laser beam used in this work. A Q-switched, frequency-doubled Nd:YAG laser (532 nm wavelength) emits a pulsed laser beam with pulse durations between 41 ns $\leq \tau_{p}\leq$ 110 ns at FWHM. The beam is shaped to a focused line. The width $W_{p}$ of the beam’s short axis (measured in the x-direction at 1/e${}^{2}$ = 0.135 of the maximum intensity *I*) is $W_{p}$ = 12 µm and the length $l_{p}$ of the long axis (in the z-direction) is $l_{p}$ = 280 µm. The intensity *I*, along the short (x-)axis, has a Gaussian distribution and a tophat distribution along the long (z-)axis.

We derive the precise value for the laser spot from the following procedure: A chemically and mechanically polished float zone Si-Wafer surface coated with an 80 nm-thick silicon nitride (SiN${}_{x}$) is used for measuring the laser spot width. The pulsed green laser irradiates the surface with different pulse energy densities with no overlap between locally melted areas. Silicon melts and evaporates during irradiation. As a consequence, the evaporating silicon surface ablates the SiN${}_{x}$ layer. Using a laser scanning microscope, we measure the ablated diameter $D_{\mathrm{abt}}$ and plot ${D_{\mathrm{abt}}}^{2}$ against the logarithm of the pulse energy density. From the slope of the linear fit to these data points, we calculate the pulse width at 1/e${}^{2}$ of the maximum intensity *I*.

Figure 2b schematically shows the used specimen for investigating the surface topography of silicon wafer after the laser process. For the purpose of getting a flat surface, a chemically and mechanically polished n-type 4-inch float zone grown silicon wafer (FZ-wafer) (Si-Mat Silicon Materials, Kaufering, Germany) is used for these experiments. A 5% hydrofluoric acid dip removes the native SiO${}_{2}$ layer on the silicon surface. The wafer is placed on an xz-translation stage. The laser beam irradiates the silicon surface with different pulse energy densities *H*. The pulse energy density *H* is calculated from the average laser power $P_{\mathrm{av}}$, the laser focus area $A_{\mathrm{spot}}$, and the pulse repetition rate *f* according to

(2) $$H\;=\;\frac{P_{\mathrm{av}}}{f\;A_{\mathrm{spot}}}.$$

We use $A_{\mathrm{spot}}$ = 12 × 280 µm${}^{2}$ and *f* = 12.5 kHz. Each laser pulse melts the silicon surface locally. For the investigation of the surface patterns, we scan the laser with a scanning speed $v_{\mathrm{scan}}$ = 300 mm/s by moving the xz-stage in the x-direction. This scanning speed is high enough to ensure no overlap between the locally melted areas on the silicon’s surface. The overlap ratio $O_{x}$ between two subsequent irradiation pulses depends on the pulse repetition rate *f*, the scanning speed $v_{\mathrm{scan}}$, and the pulse width $W_{p}$ as

(3) $$O_{x}\;=\;\left(\frac{W_{p}-\frac{v_{\mathrm{scan}}}{f}}{W_{p}}\right)\;\times \;100\%.$$

From Equation (3), using a scanning speed $v_{\mathrm{scan}}$ = 300 mm/s and a frequency *f* = 12.5 kHz, we obtain an overlap $O_{x}$ = −100%. Therefore, in x-direction, the distance between two neighboring pulses is just equal to the width of the pulses itself.

Figure 2c shows the profile of the silicon surface after resolidification. The irradiated surface is investigated using a laser scanning microscope (LSM).

### 3.2. Principles of Laser Doping

**Figure 3:**
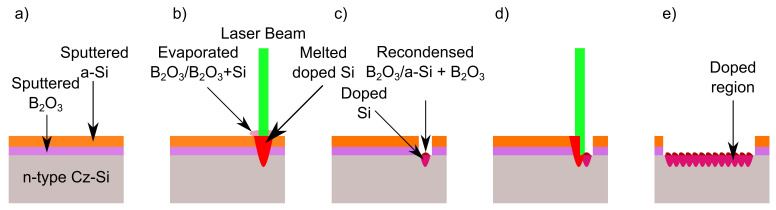
Scheme of the laser doping process: (**a**) A precursor layer stack (B${}_{2}$O${}_{3}$ covered with a-Si) covers the polished crystalline silicon. The a-Si layer protects the B${}_{2}$O${}_{3}$ layer from humidity. (**b**) A line-focused pulsed laser beam of 532 nm wavelength irradiates the precursor layer and the silicon surface. Silicon absorbs the incident laser radiation, heats up, and melts. Due to the relatively low evaporation temperature of boron oxide (1773 K [18]), the boron oxide layer evaporates during melting of silicon. Consequently, boron diffuses from the evaporated boron oxide into the molten silicon. (**c**) When the laser pulse ends, the molten volume cools down, resolidifies, and the evaporated material–B${}_{2}$O${}_{3}$ or B${}_{2}$O${}_{3}$ + Si (according to the used pulse energy density *H*)–recondenses on the relatively cold resolidified surface. The recondensed material is either boron oxide or a mixture of boron oxide and silicon. (**d**) The next laser pulse melts and evaporates the silicon surface, the precursor layer, and the recondensed, recycled material. The recycled boron atoms increase the doping level. (**e**) Finally, a boron-doped thin layer on the silicon surface is left.

Figure 3 illustrates the different phases of the laser doping process. Figure 3a shows the sample prior to irradiation. A Leybold Z550 sputtering machine (Leybold GBmbH, Cologne, Germany) deposited a two-layered precursor on a polished, chemically-cleaned 4-inch n-type Czochralski Si wafer (Cz-Si wafer) with base resistivity $\rho_{\mathrm{base}}$ = 1.5 Ω.cm and thickness $d_{\mathrm{wafer}}$ ≈ 160 µm. The machine uses a radio frequency sputtering technique (RF equals 13.56 MHz) and argon as sputtering gas. The sputtered precursor layer stack consists of a highly hygroscopic B${}_{2}$O${}_{3}$ layer [17] and an a-Si layer. The a-Si layer protects the boron oxide layer from humidity during laser processing in ambient air. The thickness of the boron oxide layer measured by profilometer is 1 nm and the a-Si layer measured by ellipsometer is 12 nm thick. The boron oxide layer serves as the dopant source for boron atoms during laser processing.

Figure 3b illustrates the doping process by a single laser pulse. For the doping processes, we use a typical scanning speed $v_{\mathrm{scan}}$ = 40 mm/s, which corresponds to an overlap $O_{x}$ = 73%. Depending on the pulse energy density *H*, the laser melts and evaporates either the B${}_{2}$O${}_{3}$ layer only or both the B${}_{2}$O${}_{3}$ layer as well as parts of the surface of the bulk silicon. Boron atoms diffuse from the boron oxide containing vapor into the liquid silicon.

Figure 3c shows the situation after one single laser shot: the doped molten silicon resolidifies and the evaporated materials are partially recondensed. In this case, they are recycled in the next shot.

Figure 3d demonstrates the situation after the second laser pulse. The subsequent laser pulse irradiates and melts the silicon surface again: the not-yet-irradiated precursor layer and the recondensed material. The recondensed, recycled materials have to be considered in the model when one aims at predicting the incorporation and diffusion of boron atoms into the liquid silicon.

Figure 3e finally delineates the doped silicon surface and the recondensed material covering it. In our laser-doping experiment, we irradiate four fields with four different pulse energy densities $H_{1}$ = 1.77 J/cm${}^{2}$, $H_{2}$ = 2.31 J/cm${}^{2}$, $H_{3}$ = 3.91 J/cm${}^{2}$, and $H_{4}$ = 4.25 J/cm${}^{2}$. After irradiation, using a diamond pen, the wafer with the four fields is cut into pieces of a size $A_{\mathrm{sample}}$ = 8 × 8 mm${}^{2}$ for the secondary ion mass spectroscopy (SIMS) measurements.

## 4. Experimental Results

This section validates the present model via a comparison with the experimental data.

**Figure 4:**
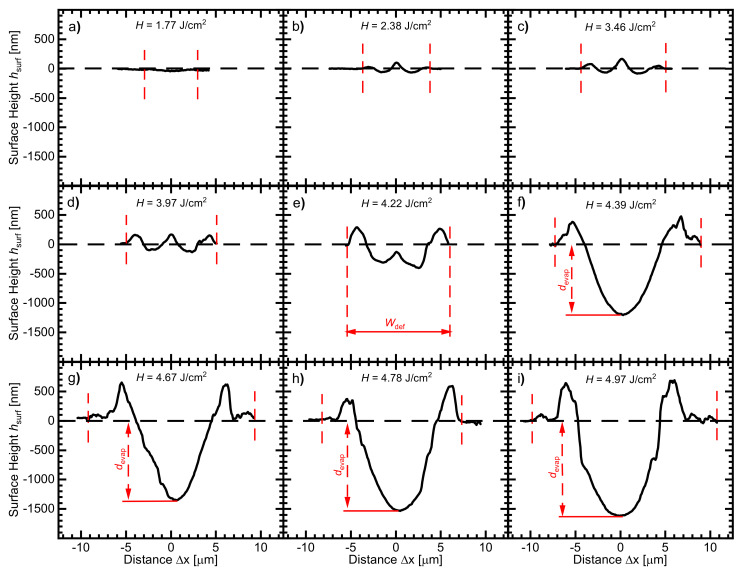
Laser scanning microscope profiles of the float zone grown silicon wafer’s surface after irradiation using single, well-separated laser shots with different laser pulse energy density *H*. The silicon surface deforms during laser irradiation, leaving behind a deformation profile of width $W_{\mathrm{def}}$. Figure 5 compares the measured deformation widths $W_{\mathrm{def}}$ with the calculated melt widths $W_{\mathrm{melt}}$ from our simulations. (**a**) For *H* = 1.77 J/cm${}^{2}$, the surface deformation is barely detectable. (**b**) At *H* = 2.38 J/cm${}^{2}$, the surface has two tiny satellite peaks with a relatively higher peak in the middle of the deformation surface profile. The tiny peaks stem from the capillary wave excited by the thermocapillary convection [19]. The peak in the middle is the result of the density anomaly of silicon during melting and resolidification [19]. (**c**,**d**) Increasing *H* boosts the thermocapillary convection within the silicon melt; as a result, the amplitude of the deformed surface peaks increases. For *H* > 2.8 J/cm${}^{2}$, as stated in Figure 1, parts of the silicon’s surface evaporate. (**e**) For *H* > 4 J/cm${}^{2}$, the depth of the molten silicon in the evaporated zone significantly increases, causing a deep tubelike capillary. Through multiple reflections of the laser in the deep capillary, the absorption of laser radiation correspondingly increases [20]. (**f**–**i**) For even higher *H*, more and more silicon evaporates. As a consequence, the height of the two satellite peaks and the depth $d_{\mathrm{evap}}$ of the missing, evaporated volume in the center increases. The deep evaporated surface hinders the creation of a middle peak via the solidification by the density anomaly like in (**e**).

Figure 4 shows the deformed surface profiles captured by the laser scanning microscope of the FZ-wafer after single-pulse irradiation using laser pulse energy densities in the range 1.77 J/cm^2^ ≤ *H* ≤ 4.97 J/cm^2^. For simplicity, not all of the captured surface profiles are included in Figure 4. However, the omitted data are finally included in the comparison of the measured and modeled data for the deformed width $W_{\mathrm{def}}$ and the evaporation depth $d_{\mathrm{evap}}$.

### 4.1. Surface Profiles

Figure 4a shows the the surface deformation for *H* = 1.77 J/cm${}^{2}$. The surface deformation is barely visible.

Figure 4b represents the surface profile when using *H* = 2.38 J/cm${}^{2}$. The deformed surface profile has two tiny satellite peaks with a relatively higher peak in the middle of the deformation surface profile. The capillary wave excited by thermocapillary convection [19] leads to the two satellite peaks. The middle peak stems from the density anomaly of silicon during melting and resolidification [19].

Figure 4c,d demonstrate that the height of the three peaks of the surface profile increase with increasing the used laser pulse energy densities *H* = 3.46 J/cm${}^{2}$ and *H* = 3.97 J/cm${}^{2}$. Here, it is worth mentioning that parts of the silicon surface evaporate when *H* exceeds 2.8 J/cm${}^{2}$, as already indicated in Figure 1. This evaporation is also confirmed by our simulations.

Figure 4e represents the deformed surface profile when increasing the used laser pulse energy density *H* = 4.22 J/cm${}^{2}$. On the one hand, the height of the two satellite peaks significantly increases. On the other hand, the height of the middle peak is significantly decreased and sunken below the original surface level. Such a high laser pulse energy density evaporates the molten silicon from even deeper, causing a deep, tubelike capillary. During silicon resolidification, the density decrease is not sufficient to compensate the evaporated depth, leaving behind a small peak in the middle.

Figure 4f shows that increasing the irradiating pulse energy density increases the height of the two satellite peaks even more. However, the middle peak disappears, as the evaporated tubelike capillary becomes deeper. The deeper-evaporated capillary in the middle of the melt hindered the density anomaly of silicon from creating a middle peak like in Figure 4e.

Figure 4g–i illustrate that not only the amplitude of the two satellite peaks but also the depth $d_{\mathrm{evap}}$ of the evaporated capillary in the middle further increases when increasing the used laser pulse energy density to be *H* = 4.22 J/cm${}^{2}$. The width of the deformation $W_{\mathrm{def}}$ (the outer distance between the satellite peaks at the level of the wafer surface) increases with the pulse energy density *H*. For *H* > 4.22 J/cm${}^{2}$, the depth of the evaporated tubelike capillary $d_{\mathrm{evap}}$ strongly increases with laser pulse energy density *H*.

**Figure 5:**
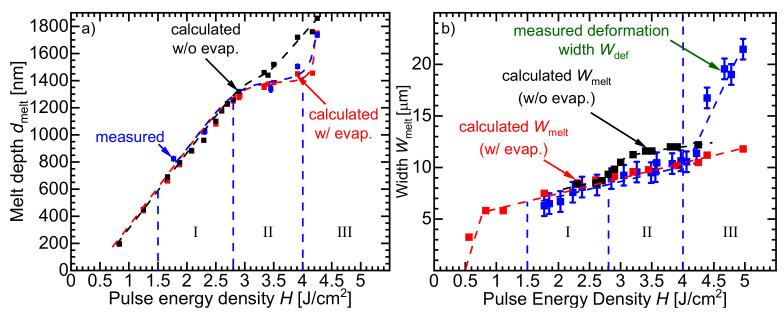
(**a**) For *H* < 2.8 J/cm${}^{2}$, in regime I, when no silicon evaporates, the calculated melt depth $d_{\mathrm{melt}}$ shows a good agreement with the simulated melt depth $d_{\mathrm{melt}}$ (extracted from the concentration profile shown later). For pulse energy density *H* > 2.8 J/cm${}^{2}$, regime II and regime III, parts of the silicon’s surface evaporate as also indicated in Figure 1. Ignoring the evaporation of silicon yields an overestimated melt depth $d_{\mathrm{melt}}$ in the simulation, because the absorbed energy is assumed to deepen the melt pool instead of evaporating the silicon’s surface. In contrast, considering the evaporation of the silicon surface leads to a much better agreement of measured and simulated melt depths $d_{\mathrm{melt}}$. (**b**) In regime I (H≤ 2.8 J/cm${}^{2}$), the calculated melt width $W_{\mathrm{melt}}$ agrees well with the measured surface deformation width $W_{\mathrm{def}}$ from the measured profiles in Figure 4. When parts of the silicon’s surface evaporate for *H* > 2.8 J/cm${}^{2}$ (in regime II), the simulation that considers the evaporation effects fits the measured values much better than the one that ignores evaporation. In regime III, for *H* > 4 J/cm${}^{2}$, the calculated melt widths deviate from the measured deformation width $W_{\mathrm{def}}$. A possible reason is that the model does not consider the enhancement of heat transfer through circulation as a result of the Marangoni effect. Another possible reason is that the absorption of laser radiation in the evaporated capillary is stronger in the x-direction than in the y-direction.

Figure 5a shows the measured melt depth $d_{\mathrm{melt}}$ extracted from the measured concentration profiles in Figure 7 in comparison to the calculated $d_{\mathrm{melt}}$ in the two cases, ignoring and taking the evaporation of silicon during irradiation into consideration. In regime I (*H* < 2.8 J/cm${}^{2}$), silicon does not evaporate, and thus, both measured and calculated melt depth $d_{\mathrm{melt}}$ agree well. However, for *H* > 2.8 J/cm${}^{2}$ (regime II), which evaporates parts of silicon’s surface, a significant overestimation occurs due to the fact that the excess absorbed energy deepens the melted volume without being consumed in the evaporation process. The estimated melt depth $d_{\mathrm{melt}}$ seems to continue increasing linearly with pulse energy density *H*. A much closer aggrement with the measured melt depth $d_{\mathrm{melt}}$ results from considering the effects of the evaporation of silicon during irradiation. A saturation-like relationship of the melted silicon depth $d_{\mathrm{melt}}$ appears to happen for 2.8 J/cm${}^{2}$ < *H* < 4 J/cm${}^{2}$. For *H* > 4 J/cm${}^{2}$ (regime III), as represented in Figure 4e, the evaporation in the middle significantly increases and thus, the total melt depth $d_{\mathrm{melt}}$ increases.

Figure 5b also compares the measured surface deformation $W_{\mathrm{def}}$ using LSM profiles in Figure 4 with the calculated melt width $W_{\mathrm{melt}}$ in cases of considering and ignoring the evaporation of silicon during irradiation. The melt width $W_{\mathrm{melt}}$ should be equal to the resolidified deformed width $W_{\mathrm{def}}$. In regime I (*H* > 2.8 J/cm${}^{2}$), there is a reasonable agreement of the calculated width of the melted surface $W_{\mathrm{melt}}$ with the measured deformed width $W_{\mathrm{def}}$. On the one hand, an overestimation of $W_{\mathrm{melt}}$ results from ignoring that parts of the silicon surface evaporate during irradiation with *H* > 2.8 J/cm${}^{2}$ (regime II). The overestimation is due to that the excess absorbed energy widens the melted volume without being consumed in the evaporation process. On the other hand, considering the evaporation effects in the model, yields a closer agreement of the melt width $W_{\mathrm{melt}}$ with the measured deformed width $W_{\mathrm{def}}$. For *H* > 4.25 J/cm${}^{2}$ (regime III), the calculated melt width $W_{\mathrm{melt}}$ is not able to correctly predict the deformed width $W_{\mathrm{def}}$. A possible reason for that is that the real or effective thermal conductivity $k_{\mathrm{Si}}$ of silicon is larger than the literature value. As a result of the enhanced absorption of radiation in the tubelike capillary due to multiple reflection, the melt mixes vigorously, which results also in an enhancement of the heat transfer process. Consequently, the melt width $W_{\mathrm{melt}}$ increases as a result of the increased effective thermal conductivity $k_{\mathrm{eff}}$ probably due to turbulent melt motion as a result of the absorbed energy. One more possible reason is the direction of laser radiation absorption in the capillary. The laser radiation is absorbed laterally on the walls of the vertical capillary. As a result, the expansion in the lateral direction increases more than that in the case of vertical absorption of radiation. Finding the effective thermal conductivity $k_{\mathrm{eff}}$ for the mixing-enhanced heat transfer is beyond the scope of this work.

Figure 5a,b validate the capability of the present model to predict the dimensions of the melt pool in the three regimes–regime I, II, and III (pulse energy density range 0 ≤H≤ 4.25 J/cm${}^{2}$). Using *H* > 4.25 J/cm${}^{2}$ in the laser doping induces defects in the doped silicon’s surface, which in turn, impairs the electronic properties of the doped area. Therefore, for the laser doping process, the pulse energy density range *H* > 4.25 J/cm${}^{2}$ is irrelevant.

### 4.2. Melting and Resolidification

The correct prediction of the time-dependent temperature is crucial for the simulation of the diffusion of the doping atoms into the liquid silicon, during the heating and cooling periods of the melt. Not only is the overall duration of these two periods important, but also the prediction of the detailed time dependence, because the diffusion coefficient of the doping atoms depends sensitively (thermally activated) on temperature, which will be discussed later.

**Figure 6:**
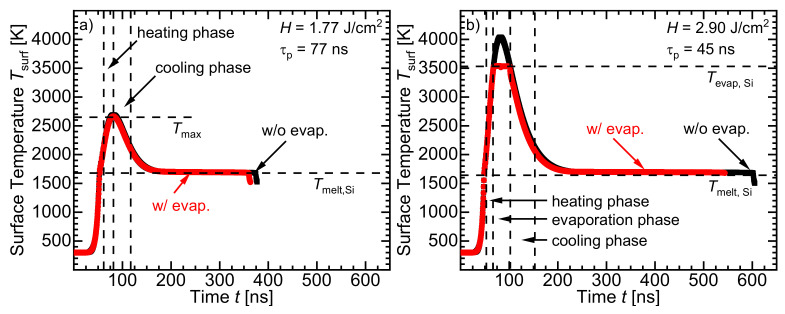
Comparison between the temperature–time curve resulted from the simulation of laser melting of silicon’s surface when considering and ignoring the evaporation effects. (**a**) Considering or ignoring the evaporation of silicon is irrelevant as no silicon evaporates at *H* = 1.77 J/cm${}^{2}$ ($\tau_{p}$ = 77 ns). Consequently, in regime I, not only the duration of heating and cooling phases, but also the maximum temperature $T_{\max}$, and hence, the diffusion duration of dopant atoms in molten silicon in both considerations is the same. (**b**) In regime II (for *H* = 2.90 J/cm${}^{2}$ with $\tau_{p}$ = 45 ns), parts of silicon’s surface evaporate. Consequently, considering the evaporation in the calculation uses the extra absorbed energy for the evaporation process rather than the overheating process. Therefore, the maximum temperature $T_{\mathrm{surf}}$ resulted from ignoring the evaporation effects in the calculation methodology is illogical as it exceeds the evaporation temperature of silicon ($T_{\mathrm{evap},\;\mathrm{Si}}$ = 3538 K [21]), and consequently, an overestimation of the calculated cool-down time occurs.

Figure 6 compares the calculated temperature–time curves resulted from ignoring and considering the evaporation effects during laser melting of the silicon’s surface. The simulation without considering evaporation of Si was done as follows: We assumed that the only existing latent heat $H_{\mathrm{Si}}$ of silicon is that for fusion *H*_melt, Si_. Similarly, we assumed also the heat capacity $C_{P_{\mathrm{Si}}}$ of liquid silicon to be valid for temperatures even beyond the nominal melting (*T*_melt, Si_ = 1687 K [16]) and evaporation *T*_evap, Si_ = 3538 K [21]) temperature of silicon.

Figure 6a represents the calculated temperature–time profile for *H* = 1.77 J/cm${}^{2}$ when considering and ignoring evaporation of silicon. At *H* = 1.77 J/cm${}^{2}$ (regime I), no evaporation of silicon takes place, and thus, the two curves agree well. The maximum reached temperature $T_{\max}$ in both cases is the same. Not only the heating phase but also the cooling phase in both cases is the same. The figure shows the heating phase to be the duration within which the temperature of the center of the molten surface $T_{\mathrm{surf}}$ increases from 2100 K to the maximum temperature $T_{\max}$. The maximum temperature $T_{\max}$ is the highest temperature reached during the whole process. The maximum temperature $T_{\max}$ increases with the used pulse energy density *H* to irradiate the surface. The cooling phase is the phase within which the temperature of the center of the melted surface $T_{\mathrm{surf}}$ decreases from the maximum temperature $T_{\max}$ down to 2100 K.

Figure 6b compares the calculated temperature–time curve when considering the evaporation of silicon during irradiation of the silicon surface with *H* = 2.90 J/cm${}^{2}$ (regime II). Ignoring the evaporation of silicon overestimates the calculated maximum temperature $T_{\max}$ and the cool-down duration. The duration that the melt needs to completely resolidify is called the cool-down duration. Considering the evaporation of silicon keeps the temperature $T_{\mathrm{surf}}$ less than or equal to the evaporation temperature *T*_evap, Si_. The excess energy absorbed evaporates the silicon, keeping the surface temperature $T_{\mathrm{surf}}\leq$ 3538 K [21]. The evaporation phase is the phase within which the temperature of the center of the molten silicon surface remains constant at the evaporation temperature of silicon *T*_evap, Si_.

### 4.3. Doping Profiles

**Figure 7:**
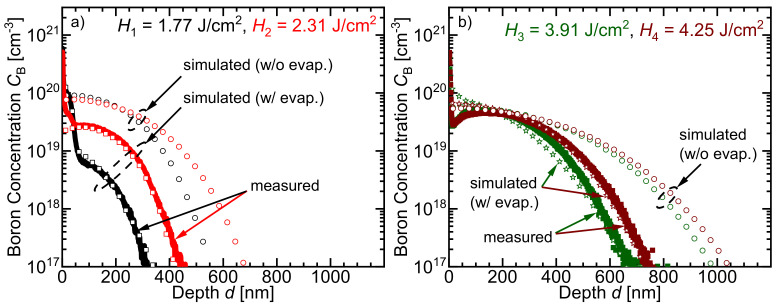
Comparison of simulated and measured secondary ion mass spectrometry concentration profiles after laser doping with a sputtered precursor layer (1 nm B${}_{2}$O${}_{3}$, and 12 nm a-Si), pulse energy densities $H_{1}$ = 1.77, $H_{2}$ = 2.31, $H_{3}$ = 3.91, and $H_{4}$ = 4.25 J/cm${}^{2}$ and pulse–pulse overlap of about 73%. (**a**) Doping at $H_{1}$ = 1.77 J/cm${}^{2}$ and $H_{2}$ = 2.31 J/cm${}^{2}$ (both in regime I) emulates the doping cases i and ii mentioned in Section 1. In this regime, the boron precursor, but not the Si itself, evaporates. Neglecting the loss of boron by evaporation of the precursor overestimates the final surface concentration and profile depth. Considering the evaporation of the precursor yields a good agreement of measured and simulated data. (**b**) For $H_{3}$ = 3.91 J/cm${}^{2}$ (regime II) and $H_{4}$ = 4.25 J/cm${}^{2}$ (regime III), not only the B${}_{2}$O${}_{3}$ but also parts of the doped melted silicon surface evaporate forming a gas doping source over the nonevaporated Si melt (doping case iii, gas/gas-melt). The simulation that considers the evaporation yields a good agreement of measured and simulated curves.

Figure 7a presents the measured and the simulated doping profiles for the specimen described in Figure 3. For doping at $H_{1}$ = 1.77 J/cm${}^{2}$ and $H_{2}$ = 2.31 J/cm${}^{2}$, both in regime I, our model simulates the doping cases melt/melt and gas/melt (cases i and ii in Section 1) well. Amorphous silicon (a-Si) melts at 1420 K [22], while boron oxide (B${}_{2}$O${}_{3}$) melts and evaporates at 723 K [18] and 1773 K [18], respectively. Subsequently, a-Si and B${}_{2}$O${}_{3}$ melt before crystalline silicon *c-Si* melts ($T_{\mathrm{melt},\;\mathrm{Si}}$ = 1687 K [16]). Thus, melt/melt doping occurs between the two melted species, a-Si and B${}_{2}$O${}_{3}$. Due to the evaporation of B${}_{2}$O${}_{3}$, the gas/melt diffusion takes place within less than 1 ns after the c-Si melts and continues until the melt resolidifies (more details are given in the Appendix C). The agreement between the measured and the simulated curves with boron oxide evaporation validates the model’s ability to simulate the melt/melt and the gas/melt doping cases. A wrong prediction of the surface concentration as well as the doping depth occurs as a result of ignoring the effects of the evaporation. However, the silicon itself does not evaporate yet in this regime. Boron oxide evaporation and expansion (concentration loss) decrease the concentration gradient at the melted silicon surface. Consequently, ignoring the evaporation process of the precursor incorporates more boron atoms in the calculated doping profile.

Figure 7b further proves the ability of our model to simulate the doping case iii (gas/gas-melt). In this regime II, for *H* > 2.8 J/cm${}^{2}$, not only boron, but also silicon evaporate, as stated by Figure 1. Doping with $H_{3}$ = 3.91 J/cm${}^{2}$ (regime II) and $H_{4}$ = 4.25 J/cm${}^{2}$ (regime III) includes all of the three abovementioned doping cases (case i, ii, and iii in Section 1). For such high pulse energy density, a part of the doped, melted silicon surface evaporates (*T*_evap, Si_ = 3538 K [21]). The previously evaporated B${}_{2}$O${}_{3}$, followed by the recently evaporated doped Si, form a gaseous doping source for the melted nonevaporated silicon (corresponding to doping case gas/gas-melt). The agreement between the simulated profiles, which consider evaporation not only for boron but also for Si, and the measured doping profiles give direct proof for the validity of our model. Evaporation of the doping source and/or parts of the doped silicon surface decreases the amount of incorporated dopant inside the resolidified material. Consequently, the doping depth is decreased. Simulations that neglect the evaporation of materials overestimate the doping profile’s height and depth.

Figure 7a,b prove that without considering the evaporation effects of not only B${}_{2}$O${}_{3}$ but also Si, it is not possible to correctly predict the doping profiles for the technologically interesting regime II. The next section describes the numerical simulation in detail.

## 5. Numerical Simulation

We exemplify our simulation by modeling the laser doping of silicon from a sputtered boron oxide precursor layer as the source for the boron doping. The boron oxide is covered by a thin a-Si layer. A purpose-developed Matlab code simulates the laser process through solving the 2-D heat equation

(4) $$\frac{\partial }{\partial x}\left(k\left(T\right)\frac{\partial T}{\partial x}\right)+\frac{\partial }{\partial y}\left(k\left(T\right)\frac{\partial T}{\partial y}\right)+\dot{q}=\rho \left(T\right)c_{p}\left(T\right)\frac{\partial T}{\partial t}$$

and the 2-D diffusion equation

(5) $$D_{c}\left(T\right)\left(\frac{d^{2}C_{B}}{dx^{2}}+\frac{d^{2}C_{B}}{dy^{2}}\right)\;=\;\frac{dC_{B}}{dt}.$$

At any given time *t*, the model calculates the temperature *T* of the elements using the input heat (absorbed radiation) $\dot{q}$, thermal conductivity k(T), heat capacity $c_{p}$, and density ρ(T) of silicon. Simultaneously, the model calculates the time-dependent boron concentration $C_{B}$ of each element using the temperature-dependent diffusion coefficient $D_{c}\left(T\right)$. The diffusion coefficient $D_{c}$(*T*) is described by the Arrhenius equation

(6) $$D_{c}\;=\;D_{0}\;exp\left(\frac{-E_{a}}{kT}\right),$$

where $D_{0}$ is the pre-exponential factor, $E_{a}$ is the activation energy, and *k* is Boltzmann’s constant.

The following subsections discuss the most important aspects of our model.

### 5.1. Numerical Discretization

**Figure 8:**
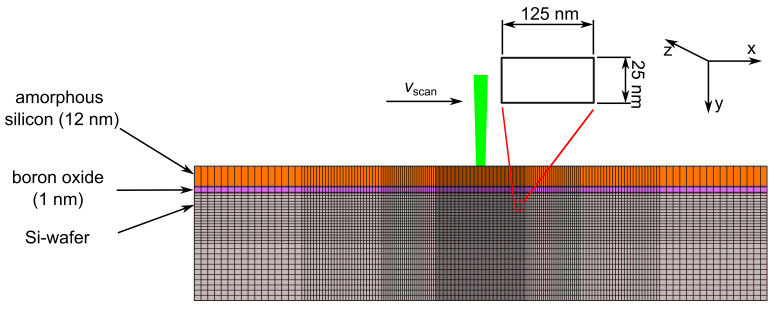
Scheme of the cross-sectional spatial discretization for numerical simulation of the silicon/precursor stack. A line-focused pulsed laser with a wavelength λ = 532 nm irradiates the precursor layer and scans the silicon surface in the x-direction. The discretization uses the adaptive grid concept to achieve increased calculation precision in the locations with high temperature gradients with a reduced calculation time and computational power [24,25,26]. The smallest element lies in the center of the laser focus and has a size of Δ*x*Δ*y* = 125 × 25 nm${}^{2}$.

Figure 8 schematically shows the spatial discretization of the considered cross-section of the silicon wafer and the precursor layer for the numerical simulation. The line-focused laser beam shown in Figure 2a irradiates the precursor layer and the silicon surface. The polished silicon surface reflects almost 37% (at wavelength λ = 532 nm and temperature *T* = 300 K) of the incident radiation [23]. Silicon has an absorption length $l_{\mathrm{abs}}$≈ 1.27 µm [23] for wavelength λ = 532 nm at temperature *T* = 300 K. Using Lambert-Beer’s law, for a wafer thickness $d_{\mathrm{wafer}}$ = 160 µm, the laser energy transmitted through the wafer is negligibly small. Consequently, heating up and melting of the silicon surface are results of the absorption of the nonreflected part. The tophat distribution of the laser intensity *I* in the long axis minimizes the variation of the temperature of the heated silicon along the z-axis. Consequently, we model the laser doping process in two dimensions, x (scanning direction) and y (direction of wafer-surface normal). All the material properties used in this work are given in the Appendix A.

An adaptive grid divides the considered volume into different sizes of elements. In the center of each element, a mesh point exists. The center of the observed cross section has finer-sized elements. The mesh size increases in the lateral direction and in the depth of the silicon wafer. The laser beam irradiates the surface in the center of the fine-size area for the numerical simulation. The advantage of adapting the mesh size is to achieve an accurate calculation of temperature and concentration in the region where the laser melts the silicon without significantly increasing the computational power. A detailed description of the implementation of the adaptive grid concept is presented in references [24,25,26].

The smallest element in the simulation area exists exactly underneath the center of the laser focus and has a size ΔxΔy = 25 × 125 µm${}^{2}$. In order to get a precise but fast simulation of the doping process, we optimized the mesh size. The biggest mesh size with which we got the same result as for the finer mesh sizes was 125 × 25 nm${}^{2}$. A smaller mesh size does not increase the precision, but only significantly increases the computing time. A bigger mesh size impairs the accuracy of the result accordingly. With the chosen mesh size, a simulation of a complete doping profile takes less than 7 h.

### 5.2. Laser Absorption

The precursor layer system does not contribute to the absorption of the laser radiation. The absorption length $l_{\alpha }$ of amorphous silicon for radiation with wavelength λ = 532 nm at room temperature (300 K) is around $l_{\alpha }$ = 460 nm (absorption coefficient $\alpha_{a-\mathrm{Si}}$ = 21.88 × 10${}^{3}$ cm${}^{-}$${}^{1}$ [27]). Using the Lambert-Beer law, the absorbed portion of the incident laser radiation in the 12 nm-thick a-Si layer is less than 2.5% of the incident radiation energy. Therefore, the model considers the precursor layer system as transparent for the incident laser beam.

Due to the laser pulse, the surface of the Si wafer heats up and also heats up the boron oxide. The boron oxide evaporates and boron atoms diffuse into the molten silicon surface.

For *H* > 4 J/cm${}^{2}$, the evaporated tubelike capillary at the center of the melted silicon enhances the absorption of the laser radiation. The absorption enhancement stems from multiple reflections of radiation within the evaporated tubelike capillary. In Figure 4e–i, during the irradiation with *H* > 4 J/cm${}^{2}$, the depth of the evaporated capillary in the center of the silicon melt is deep enough to cause multiple reflections of the incident laser radiation [20], and thus, enhance the absorption of laser in the evaporated volume. The simulation considers the enhanced absorption of laser inside the evaporated capillary by a locally reduced reflection $R_{\mathrm{Si},\;l}$ of the melted surface in the evaporated capillary.

**Figure A1:**
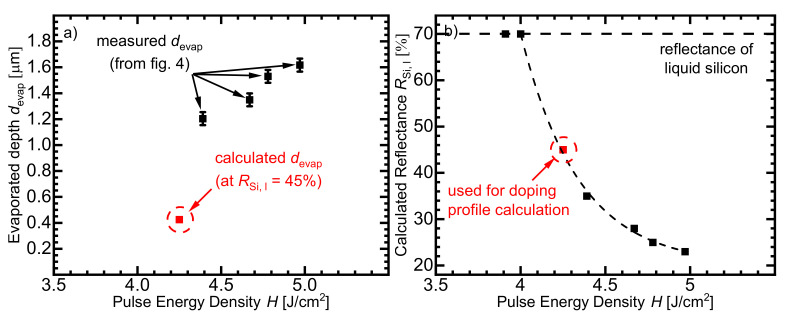
(**a**) Measured depths $d_{\mathrm{evap}}$ of the evaporated capillary from the deformation profiles of Figure 4 for *H* > 4.39 J/cm${}^{2}$. When *H* increases, the depth $d_{\mathrm{evap}}$ of the resolidified capillary increases. Direct measuring of $d_{\mathrm{evap}}$ for *H* = 4.22 J/cm${}^{2}$ is not possible after crystallization, therefore, the model calculated $d_{\mathrm{evap}}$ using the $R_{\mathrm{Si},\;l}$ value obtained from the fit curve in Figure A1b. (**b**) Within the evaporated capillary, multiple-reflection-enhanced absorption of laser radiation takes place for *H* > 4 J/cm${}^{2}$ via a reduction of the surface reflectance $R_{l-\mathrm{Si}}$ of the molten Si within the evaporated capillary. For each *H* at varied $R_{\mathrm{Si},\;l}$, we compare the calculated depth of the evaporated capillary $d_{\mathrm{evap}}$ with that measured from Figure 4f–i until the correct match at the correct calculated $R_{\mathrm{Si},\;l}$ occurs. For pulse energy densities *H* < 4 J/cm${}^{2}$, the reflectance of the liquid Si surface in the evaporated volume $R_{l-\mathrm{Si}}$ is equal to the literature value $R_{\mathrm{Si},\;l}$ = 70% [23].

The depth of the evaporated capillary $d_{\mathrm{evap}}$ in the deformed surface profile for *H* > 4.0 J/cm${}^{2}$, shown in Figure 4, gives a clue about the depth of the evaporated capillary. In the modeling, we use the measured $d_{\mathrm{evap}}$ to calculate the equivalent surface reflectance $R_{\mathrm{Si},l}$ of the melted silicon surface in the evaporated capillary. The comparison between the calculated evaporation depth $d_{\mathrm{evap}}$ for different values of $R_{\mathrm{Si},l}$ with the measured $d_{\mathrm{evap}}$ enables us to adjust the $R_{\mathrm{Si},\;l}$ value for each pulse energy density *H*. For *H* = 4.22 J/cm${}^{2}$, no measurable $d_{\mathrm{evap}}$ appears in Figure 4e. Therefore, we used the fit curve appearing in Figure A1b to find the corresponding $R_{\mathrm{Si},\;l}$-value. The reflectance $R_{\mathrm{Si},\;l}$ for all *H* values is lower than the reported literature value (70% at T≥ $T_{{\mathrm{melt}}_{\mathrm{Si}}}$ [23]). Appendix B lists the calculated reflectance $R_{\mathrm{Si},l}$ versus pulse energy density *H*.

**Figure 9:**
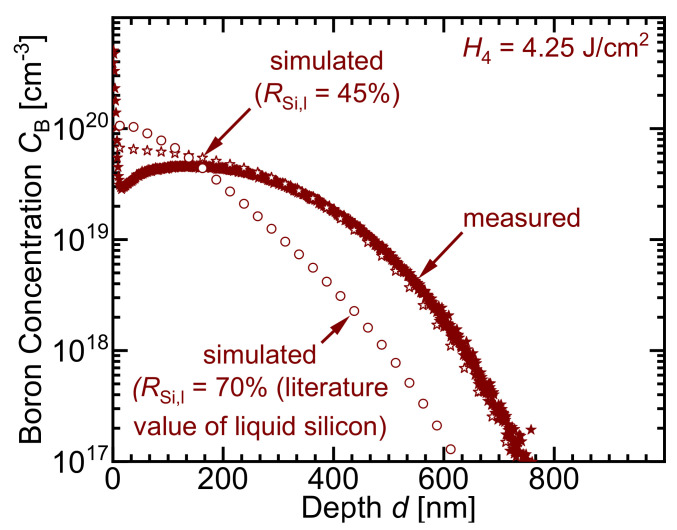
With the literature value for the reflectance $R_{\mathrm{Si},l}$ of the molten silicon surface, the simulation underestimates the doping profile. Using a lower reflectance $R_{\mathrm{Si},l}$ value of the molten silicon surface accurately fits the concentration profile.

According to Figure 9, the best fit for the concentration profile at *H* = 4.25 J/cm${}^{2}$ is obtained when a lower melted surface reflectance $R_{\mathrm{Si},l}$ is used in the evaporated capillary during the evaporation phase. For *H* = 4.25 J/cm${}^{2}$, using only the literature value of the reflectance of the molten silicon surface (70% [23]) underestimates the doping profile depth and overestimates the surface concentration. Using a reflectance $R_{\mathrm{Si},l}$ = 45% during the evaporation phase better predicts the correct concentration profile.

### 5.3. Heat Transfer

On the surface of the solid or liquid Si, convective heat transfer to the air is neglected for the following reasons: At the melting temperature $T_{{\mathrm{melt}}_{\mathrm{Si}}}$ = 1687 K [16] of silicon, the thermal conductivity $k_{\mathrm{Si},\;s}$ = 22.8 W/(m K) of solid silicon and $k_{\mathrm{Si},l}$ = 62 W/(m K) [28] of liquid silicon are significantly higher than the thermal conductivity $k_{\mathrm{air}}$ = 0.01 − 0.08 W/(m K) [29] of air.

To account for overheating (during melting with laser of pulse duration $\tau_{p}$ = 42 ns) and undercooling (during resolidification), the model uses the melting and resolidification temperatures with 20 K differences from the literature values [30,31], which is a reasonable value for the solid/liquid interface velocity 8 m/s < $v_{\mathrm{int}}$ < 10 m/s. Thus, the melting temperature in the model equals $T_{{\mathrm{melt}}_{\mathrm{Si}}}$ + 20 K and the resolidification temperature equals $T_{{\mathrm{melt}}_{\mathrm{Si}}}$ = $T_{{\mathrm{melt}}_{\mathrm{Si}}}$− 20 K.

On the time scale, upon laser irradiation, the first diffusion takes place between the melted a-Si layer ($T_{{\mathrm{melt}}_{a-\mathrm{Si}}}$ = 1420 K [22]) and the melted boron oxide layer ($T_{{\mathrm{melt}}_{B_{2}O_{3}}}$ = 723 K [18]). Later, after the c-Si melts ($T_{{\mathrm{melt}}_{\mathrm{Si}}}$ = 1687 K [16]), B-atoms diffuse only from evaporated B${}_{2}$O${}_{3}$ into the molten c-Si surface because the energy needed to heat up and evaporate a 1 nm-thick B${}_{2}$O${}_{3}$ element equals the energy needed to melt 1.5 nm of c-Si element. The detailed calculation is given in the Appendix. The finest silicon element considered in the numerical discretization in Figure 8 has a depth Δy = 25 nm. Consequently, the B${}_{2}$O${}_{3}$ element evaporates as soon as the underlying c-Si element melts.

**Figure 10:**
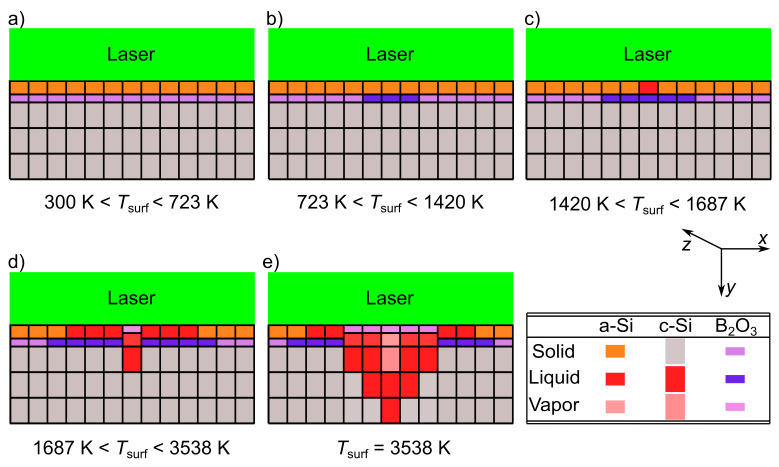
The different phases of the crystalline silicon (c-Si) wafer cross-section and the precursor layers during laser irradiation. The spatial discretization represented in Figure 8 divides the irradiated system into elements. (**a**) The surface temperature $T_{\mathrm{surf}}$ at the center of the beam increases from 300 K until below the melting temperature $T_{{\mathrm{melt}}_{B_{2}O_{3}}}$ = 723 K [18] of boron oxide. (**b**) When $T_{\mathrm{surf}}\geq$ 723 K, the B${}_{2}$O${}_{3}$ melts, while the a-Si layer and c-Si surface are still solid. (**c**) Melt/melt diffusion between the melted B${}_{2}$O${}_{3}$ and the melted a-Si starts when a-Si melts at $T_{\mathrm{surf}}\geq$ 1420 K [22]. (**d**) At $T_{\mathrm{surf}}\;\geq$ $T_{{\mathrm{melt}}_{\mathrm{Si}}}$ = 1687 K [16], the c-Si melts and the B${}_{2}$O${}_{3}$ evaporates. A matrix reordering of the melted and the evaporated elements takes place; evaporated elements go up, melted elements go down. (**e**) For pulse energy density *H* sufficient to evaporate silicon, the surface temperature $T_{\mathrm{surf}}$ reaches $T_{{\mathrm{evap}}_{\mathrm{Si}}}$ = 3538 K [21]. At $T_{\mathrm{surf}}$ = $T_{{\mathrm{evap}}_{\mathrm{Si}}}$ = 3538 K [21], a-Si and c-Si elements evaporate and gas/gas-melt diffusion of boron atoms occurs.

Figure 10 shows the different phases that occur during laser irradiation on the surface of the crystalline silicon (c-Si) wafer with the precursor layer (12 nm a-Si + 1 nm B${}_{2}$O${}_{3}$). The irradiated system is represented in the form of the discretized elements shown in Figure 8. In Figure 10a, at the center of the laser beam, where the elements with the finest size in Figure 8 exist, the absorbed laser power increases the surface temperature $T_{\mathrm{surf}}$ from room temperature until below the melting temperature $T_{{\mathrm{melt}}_{B_{2}O_{3}}}$ = 723 K [18] of boron oxide. In Figure 10b, the boron oxide melts at $T_{\mathrm{surf}}\geq T_{{\mathrm{melt}}_{B_{2}O_{3}}}$ = 723 K, while the a-Si layer and c-Si surface are still solid. Figure 10c shows the start of the melt/melt diffusion between the melted boron oxide and the melted a-Si when $T_{\mathrm{surf}}$ reaches the melting temperature $T_{{\mathrm{melt}}_{a-\mathrm{Si}}}$ = 1420 K [22] of a-Si. More boron oxide melts as $T_{\mathrm{surf}}$ increases. When $T_{\mathrm{surf}}$ reaches the melting temperature $T_{{\mathrm{melt}}_{\mathrm{Si}}}$ = 1687 K [16] of c-Si, the c-Si acquires the latent heat of fusion $H_{{\mathrm{fusion}}_{\mathrm{Si}}}$ and melts. In the same time, B${}_{2}$O${}_{3}$ takes up the latent heat of vaporization $H_{{\mathrm{vap}}_{B_{2}O_{3}}}$ and evaporates, as shown in Figure 10d. As soon as B${}_{2}$O${}_{3}$ evaporates, in the simulation, a reordering of the matrix elements takes place: the evaporated elements go up and the melted elements move down. Figure 10e shows the case for *H* being high enough to evaporate the silicon ($T_{{\mathrm{evap}}_{\mathrm{Si}}}$ = 3538 K [21]). In this case, the surface temperature reaches $T_{\mathrm{surf}}$ = $T_{{\mathrm{evap}}_{\mathrm{Si}}}$ = 3538 K [21]. At $T_{\mathrm{surf}}$ = $T_{{\mathrm{evap}}_{\mathrm{Si}}}$ = 3538 K [21], the a-Si layer and a portion of the superficial c-Si elements evaporate and gas/gas-melt diffusion takes place.

### 5.4. Diffusion

Depending on the maximum temperature in the melt, the pre-exponential factor $D_{0}$ (in Equation (6)) of the diffusion coefficient of boron in liquid silicon $D_{c_{B}}$ has two values. The temperature $T_{\mathrm{surf}}$ is the maximum temperature existing in the melt. At $T_{\mathrm{surf}}$ < 2100 K, the pre-exponential factor $D_{0}$ equals 2.7 × 10${}^{-4}$ cm${}^{2}$/s (the literature value in Ref. [32]), and at $T_{\mathrm{surf}}$ > 2100 K, $D_{0}$ equals 8 × 10${}^{-4}$ cm${}^{2}$/s (the reported value in the work of Lill et al. [13]). Appendix D presents a detailed calculation, showing that the diffusion coefficient $D_{c_{B}}$ of boron in silicon must increase during the laser doping process.

**Figure 11:**
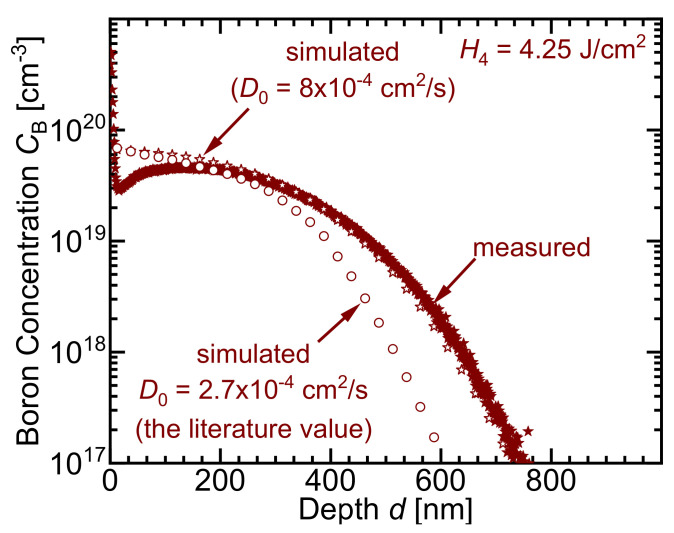
The best fit to the measured doping profiles occurs when using a higher pre-exponential factor $D_{0}$ value depending on the maximum temperature $T_{\mathrm{surf}}$. For $T_{\mathrm{surf}}\geq$ 2100 K, the higher pre-exponential diffusion coefficient $D_{0}$ = 8 × 10${}^{-4}$ cm${}^{2}$/s is used. After the surface temperature cools down again to $T_{\mathrm{surf}}\leq$ 2100 K, the literature value $D_{0}$ = 2.7 × 10${}^{-4}$ cm${}^{2}$/s is used again [32]. Exemplary, using only the literature value of $D_{0}$ underestimates the profile depth. A better fit is only achievable when using the combination of both values of $D_{0}$ with surface temperature $T_{\mathrm{surf}}$ as the condition.

Figure 11 also confirms that the best fit of the measured doping profiles is obtained when two values for the pre-exponential factor $D_{0}$ are used: $D_{0,\;1}$ = 8 × 10${}^{-4}$ cm${}^{2}$/s (the reported value in the work of Lill et al. [13]) for temperatures $T_{\mathrm{surf}}$ > 2100 K; $D_{0,\;2}$ = 2.7 × 10${}^{-4}$ (the literature value in reference [32]) for temperatures $T_{\mathrm{melt},\;\mathrm{Si}}$ < $T_{\mathrm{surf}}$ < 2100 K.

We choose 2100 K as the transition value for the following reason: this temperature is reached at the surface for H≤ 1.2 J/cm${}^{2}$, which results in no visible surface deformation after irradiation. No surface deformation means that either the surface did not melt at all, or it melted without circulation. For this case, we take the literature value for $D_{0}$. For higher *H* > 1.2 J/cm${}^{2}$, the silicon surface deforms as a result of the irradiation, indicating that Marangoni convection took place. This convection in turn increases the diffusion with a consequence of a higher $D_{0}$. For that reason, the chosen transition temperature is the calculated maximum temperature $T_{\max}$ in the center of the melt when a pulse energy density *H* = 1.2 J/cm${}^{2}$ is used.

### 5.5. Recondensation

**Figure 12:**
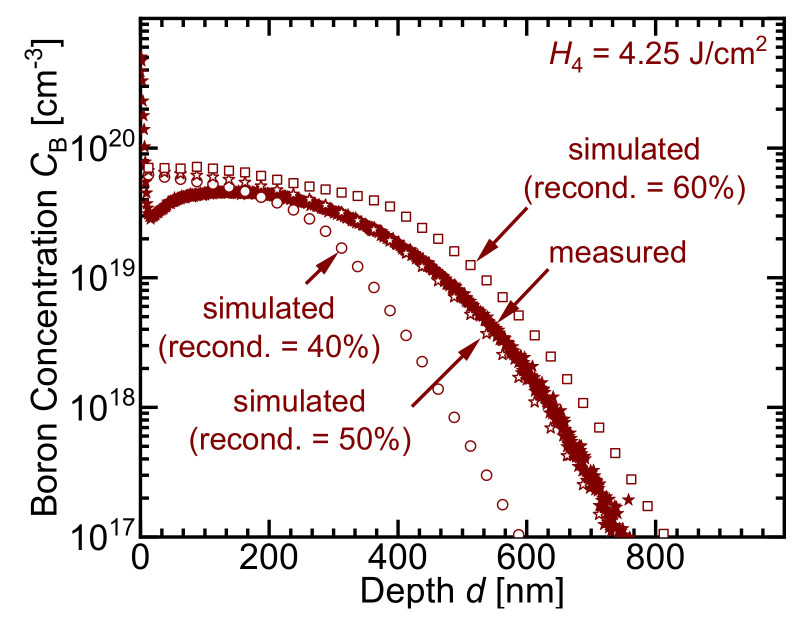
A 40% and a 60% recondensation portion of the evaporated materials after each laser pulse yield an underestimation and overestimation of the concentration profiles, respectively. The best fit results from using a 50% recondensation portion.

After each laser pulse, parts of the evaporated material (percursor material and for high laser energies also Si) recondense. Figure 12 demonstrates that the calculated doping profiles are fit best assuming a 50% recondensation of the evaporated materials after each pulse. For 40% recondensation, an underestimation of the calculated doping profile depth occurs. For 60%, an overestimation of the simulated doping profile occurs. For the same recondensation of 50%, Eisele and Köhler [30] also obtained the best fits for the measured doping profiles when using phosphosilicate glass as a precursor for laser doping.

For *H* > 4 J/cm${}^{2}$, where a deep tubelike capillary of silicon evaporates, the evaporated silicon and boron oxide recondense as a boron-rich a-Si layer. Considering that 50% of the evaporated materials recondense, the recondensed layer, under certain experimental conditions, reaches a thickness of more than 200 nm. A thickness of more than 25 nm (the thickness of the finest crystalline silicon element in Figure 8) contributes to the absorption of laser radiation and heat transfer calculations. For correct calculation of energy absorption and heat transfer, the relatively thick recondensed layer is divided into a corresponding number of sublayers, each having a thickness of Δy = 25 nm.

## 6. Discussion and Conclusions

The present model numerically simulates the laser doping process of silicon with a low evaporation temperature precursor and also for high pulse energy densities *H*, where silicon evaporates. Previous models were either limited to precursor layers with a high melting and evaporation temperature [13], or they did not work when silicon evaporates due to high laser pulse energy densities [13,14,30]. Some models can only simulate laser-processing with a cw-laser [15]. A detailed comparison of simulated with experimental results of melt width and depth validates the capability of our model to predict the dimensions of the melt pool in the three regimes, regimes I, II, and III, until *H* = 4.25 J/cm${}^{2}$. The very good agreement between the calculated and the measured doping profiles for the investigated wide pulse energy density 1.77 J/cm${}^{2}$≤H≤ 4.25 J/cm${}^{2}$ range proves that the correct predictions of the doping profiles are only achievable when considering the evaporation effects of the precursor layer and Si. Finally, we showed that the best fits of the doping profiles occur when 50% recondensation of the evaporated materials, reduced reflectance in the evaporated capillary during the evaporation phase, and a higher diffusion coefficient are used.

## Figures and Tables

**Figure A2:**
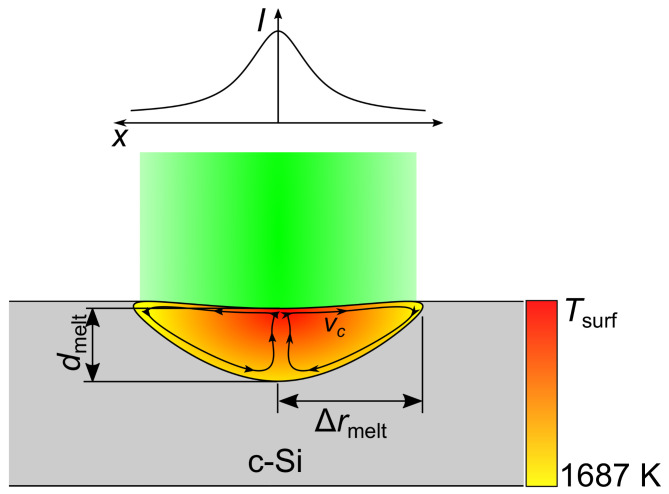
Marangoni convection: The colors represent the temperature distribution inside the molten volume. Red color represents the maximum and yellow represents the melting temperature (the coldest liquid silicon). Due to the Gaussian distribution of laser intensity *I* in the x-direction, the hottest temperatures in the melt occur in the center of the molten surface. The coldest lie at the boundary between molten and solid silicon. The surface tension gradient induced by the temperature gradient mixes the melt. The temperature distribution in the melt is symmetric around the pool axis. As a result, the melt moves by circulating from the hotter center to the colder edges. This phenomenon is also called the *thermocapillary effect*.

**Figure A3:**
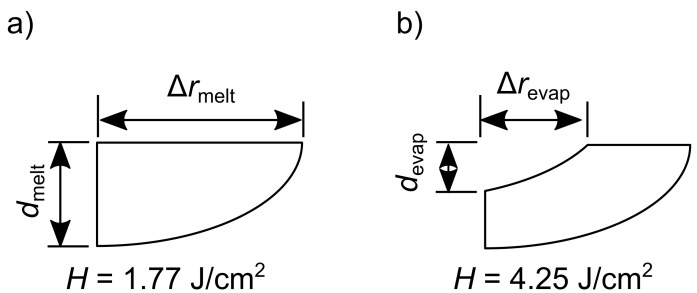
The circumference of the half melt pool is calculated according to the geometrical shape of the melt pool. The whole melt pool is approximated by a half ellipse. (**a**) At *H* = 1.77 J/cm${}^{2}$, the half melt pool is a quarter of an ellipse. (**b**) At *H* = 4.25 J/cm${}^{2}$, the half melt pool is approximated by two concentric ellipses, in order to consider the evaporated portion.

## Data Availability

Data is contained within the article.

## Appendix A. Material Properties

The temperature-dependent optical properties like refractive index $n_{\mathrm{Si}}$ and absorption coefficient $\alpha_{\mathrm{Si}}$ of solid silicon used in this work are taken from Ref. [23]. The optical properties of liquid silicon stem from Ref. [33]. A summary of the material properties used in this work is shown in Table A1 and Table A2.

**Table A1:** Properties of crystalline silicon.

Property	Solid	Liquid	Unit	Reference
Density $\rho_{\mathrm{Si}}$	$2330-2.19\times 10^{-2}\;T$	$2540-2.19\times 10^{-2}\;T-1.21\times 10^{-5}\;T^{2}$	$\left[\frac{\mathrm{kg}}{m^{3}}\right]$	[28,34]
Thermal conductivity $k_{\mathrm{Si}}$	$22.23+422.52\times exp\left(\frac{-T}{255.45}\right)$	62	$\left[\frac{W}{m\;K}\right]$	[28]
Heat capacity $c_{p_{\mathrm{Si}}}$	$352.43+1.78\;T-2.21\times 10^{-3}\;T^{2}+1.3\times 10^{-6}\;T^{3}$	1021.84	$\left[\frac{J}{\mathrm{kg}\;K}\right]$	[28]
Latent Heat $H_{\mathrm{Si}}$	1802 (Melting)	16,207 (Vaporization)	$\left[\frac{\mathrm{kJ}}{\mathrm{kg}}\right]$	[16,28]

**Table A2:** Properties of precursor layer.

Property	Solid	Liquid	Unit	Reference
Melting Temperature $T_{{\mathrm{melt}}_{a-\mathrm{Si}}}$	1420 (Melting)	–	[K]	[22]
Melting Temperature $T_{{\mathrm{melt}}_{B_{2}O_{3}}}$	723 (Melting)	–	[K]	[18,35]
Evaporation Temperature $T_{{\mathrm{evap}}_{B_{2}O_{3}}}$	–	1773	[K]	[18]
Latent Heat $H_{B_{2}O_{3}}$	0.317 (Melting)	4.308 × 10${}^{6}$ (Vaporization)	$\left[\frac{J}{\mathrm{kg}}\right]$	[18]

## Appendix B. Aspect: Laser Absorption

Figure A1a represents the measured depth $d_{\mathrm{evap}}$ of the evaporated capillary tubes shown in Figure 4f–i. The higher the pulse energy density *H* is, the deeper the evaporated depth $d_{\mathrm{evap}}$ becomes. Although silicon evaporates for *H* > 2.8 J/cm${}^{2}$ (as stated in Figure 1), the evaporated capillaries firstly show up for *H* > 4.0 J/cm${}^{2}$ (starting from the profile in Figure 4e). For 2.8 J/cm${}^{2}$ < *H* < 4.39 J/cm${}^{2}$ range, the middle peak in the center of the deformed profile resulting from the silicon density anomaly [19] does not allow an exact measurement of the evaporated depth $d_{\mathrm{evap}}$. From the fit curve in Figure A1b, the reduced calculated equivalent reflectance at *H* = 4.22 J/cm${}^{2}$ and $H_{4}$ = 4.25 J/cm${}^{2}$ is $R_{\mathrm{Si},\;l}$ = 45%, which gives a calculated $d_{\mathrm{evap}}$ = 425 nm at $H_{4}$ = 4.25 J/cm${}^{2}$.

Figure A1b presents the calculated reduced reflectance $R_{\mathrm{Si},\;l}$ of the molten silicon surface in the evaporated capillary. Due to multiple reflections, the absorption in the capillary increases, therefore, the overall reflection $R_{\mathrm{Si},\;l}$ of the Si surface within the capillary decreases. In comparison with the measured depth $d_{\mathrm{evap}}$, simulating with the literature value of $R_{\mathrm{Si},\;l}$ (70% at T≥ $T_{\mathrm{melt},\;\mathrm{Si}}$ [23]) yields smaller values for the calculated depths $d_{\mathrm{evap}}$. The measured depth $d_{\mathrm{evap}}$ serves as a target value in the determination process of the reduced reflectance $R_{\mathrm{Si},\;l}$ for each pulse energy density *H*. The model varies the calculated reduced reflectance $R_{\mathrm{Si},\;l}$ until the calculated depth $d_{\mathrm{evap}}$ matches the directly measurable capillary depths $d_{\mathrm{evap}}$ for *H* > 4.39 J/cm${}^{2}$. For *H* < 4.0 J/cm${}^{2}$, the reflectance $R_{\mathrm{Si},\;l}$ is $R_{\mathrm{Si},\;l}$ = 70%. From the fit curve plotted, we determine the calculated reduced reflectance $R_{\mathrm{Si},\;l}$ for *H* = 4.25 J/cm${}^{2}$, since we are unable to measure $d_{\mathrm{evap}}$ from the profile in Figure 4e.

## Appendix C. Aspect: Heat Transfer

The latent heat of fusion of Si is $H_{\mathrm{fusion},\;\mathrm{Si}}$ = 1802 J/g [16] and the latent heat of evaporation B${}_{2}$O${}_{3}$ is $H_{\mathrm{evap},\;B_{2}O_{3}}$ = 4308 J/g [18]. The density and heat capacity of liquid B${}_{2}$O${}_{3}$ at evaporation temperature $T_{\mathrm{evap},\;B_{2}O_{3}}$ = 1773 K [18] are $\rho_{B_{2}O_{3},\;l}$ = 1.498 g/cm${}^{3}$ [36,37] and $c_{p_{B_{2}O_{3},\;l}}$ = 0.4472 J/(g K) [38]. The density of solid silicon at melting temperature $T_{\mathrm{melt},\;c-\mathrm{Si}}$ = 1687 K [16] is $\rho_{\mathrm{Si},\;s}$ = 2.293 g/cm${}^{3}$ [34]. The required energy per unit area $E_{B_{2}O_{3}}$ to heat up and evaporate 1 nm of B${}_{2}$O${}_{3}$ per unit area is

(A1) $$E_{B_{2}O_{3}}\;=\;\rho_{B_{2}O_{3},\;l}\;d_{B_{2}O_{3}}\;\left[c_{p_{B_{2}O_{3}}}\;\left(1773-1687\right)+H_{\mathrm{evap},\;B_{2}O_{3}}\right]=651\;{\mu J/\mathrm{cm}}^{2}.$$

The required energy per unit area $E_{\mathrm{Si}}$ to heat up and evaporate 25 nm of c-Si per unit area is

(A2) $$E_{\mathrm{Si}}\;=\;\rho_{\mathrm{Si},\;s}\;d_{\mathrm{Si}}\;H_{\mathrm{melt},\;\mathrm{Si}}=10300\;{\mu J/\mathrm{cm}}^{2}.$$

Therefore, the energy needed to heat up and evaporate a 1 nm-thick B${}_{2}$O${}_{3}$ equals the energy needed to melt 1.5 nm of c-Si. Consequently, the 1 nm-thick B${}_{2}$O${}_{3}$ element evaporates as soon as a 25 nm-thick c-Si element melts. Thus, the diffusion process takes place between boron oxide vapor and the molten crystalline silicon surface.

## Appendix D. Aspect: Diffusion

The best fits of the measured concentration profiles of boron after laser doping are obtained when two values for the pre-exponential factor $D_{0}$ are used: $D_{0,\;1}$ = 8 × 10${}^{-4}$ cm${}^{2}$/s (the reported value in the work of Lill et al. [13]) for temperatures $T_{\mathrm{surf}}$ > 2100 K; $D_{0,\;2}$ = 2.7 × 10${}^{-4}$ (the literature value in reference [32]) for temperatures $T_{\mathrm{melt},\;\mathrm{Si}}$ < $T_{\mathrm{surf}}$ < 2100 K.

As a result of the Gaussian distribution of laser intensity *I* in the x-direction, a temperature gradient results between the hot center and the colder edges. The edges are just melted, or in other words, are at melting temperature. The temperature gradient induces a surface tension gradient, which pulls the hotter (molten) silicon in the center towards the colder edges. The surface tension gradient in the melt induces a circulatory motion in the melt. This phenomenon is called the *thermocapillary effect* or *Marangoni convection* [19].

Figure A2 schematically shows the circulatory motion induced in the melt as a result of the Marangoni convection. The element of the highest temperature in the melted silicon is the surface element at the center of the melt. The coldest elements (just melted) lie at the boundary between the liquid and solid silicon. The surface tension σ of the molten silicon depends on the temperature *T*. The temperature gradient across the molten silicon surface causes a surface tension gradient ∂σ/∂T (≈86 × 10${}^{-6}$ N/(m K) [39]). The melt circulates from the hot center towards the colder edges as a result of the temperature-gradient-induced surface tension gradient. The circulatory motion enhances the mass transport inside the melted volume, and thus, leads to an apparently increased “effective” pre-exponential factor $D_{0,\;1}$. The literature values of $D_{0}$ are obtained from a silicon melt of a homogeneous temperature distribution. The reported values of $D_{0}$ are 2.4 ± 0.7 × 10${}^{-4}$ cm${}^{2}$/s [40] and 2.7 × 10${}^{-4}$ cm${}^{2}$/s [32].

The equation for calculating the circulation velocity $v_{c}$ [41] is

(A3) $$v_{c}\;=\;\frac{\partial \sigma }{\partial T}\frac{\left(T_{\mathrm{surf}}-T_{{\mathrm{melt}}_{\mathrm{Si}}}\right)}{\Delta r_{\mathrm{melt}}}\frac{d_{\mathrm{melt}}}{\eta },$$

where $\Delta r_{\mathrm{melt}}$ is the length, over which the temperature gradient extends, and η is the dynamic viscosity of silicon (η≈ 0.75 mN s/m${}^{2}$ [42]).

Figure A3 shows the approximated shape of half of the melt pool. The circumference is calculated according to the closest geometrical shape. The melt pools are simulated by ellipses. Figure A3a demonstrates that, at *H* = 1.77 J/cm${}^{2}$, the melt pool is a quarter of an ellipse. Its perimeter is $L_{\mathrm{melt},\;\mathrm{total}}$ = ($a_{\mathrm{melt}}$ + $b_{\mathrm{melt}}$ )(1 + π/4), where $a_{\mathrm{melt}}$ = $\Delta r_{\mathrm{melt}}$ = 3 µm and $b_{\mathrm{melt}}$ = $d_{\mathrm{melt}}$ = 0.75 µm. Consequently, the total circumference of the half melt pool at *H* = 1.77 J/cm${}^{2}$ is $L_{\mathrm{melt},\;\mathrm{total}}\approx$ 6.7 µm. At *H* = 4.25 J/cm${}^{2}$, the half of the melt pool is approximated by two concentric ellipses. The inner ellipse represents the evaporated portion. The circumference of the pictured half of the melt pool is $L_{\mathrm{melt},\;\mathrm{total}}$ = ($a_{\mathrm{melt}}$ + $b_{\mathrm{melt}}$)(1 + π/4) + ($a_{\mathrm{evap}}$ + $b_{\mathrm{evap}}$)(π/4 − 1), where $a_{\mathrm{melt}}$ = $\Delta r_{\mathrm{melt}}$ = 5.25 µm, $b_{\mathrm{melt}}$ = $d_{\mathrm{melt}}$ = 1.73 µm, $a_{\mathrm{evap}}$ = $\Delta r_{\mathrm{evap}}$ = 2.5 µm, and $b_{\mathrm{evap}}$ = $d_{\mathrm{evap}}$ = 0.48 µm. Therefore, the total circumference of the half melt pool at *H* = 4.25 J/cm${}^{2}$ is $L_{\mathrm{melt},\;\mathrm{total}}\approx$ 11.8 µm.

The simulation and validation of the time-dependent temperature during the heating and cooling in Section 4.2 allow us to calculate the maximum temperature reached during the irradiation. Furthermore, we calculate the heating, cooling, and evaporation durations $t_{\mathrm{heating}}$, $t_{\mathrm{cooling}}$, and $t_{\mathrm{evap}}$ as well as the melt depths $d_{\mathrm{melt}}$ to estimate the circulation velocity $v_{c}$ of the molten silicon. For a pulse energy density *H* = 1.77 J/cm${}^{2}$, the maximum temperature $T_{\max}$ reached during the irradiation is $T_{\max}$ = 2660 K and, for *H* = 4.25 J/cm${}^{2}$, the maximum temperature $T_{\max}$ reaches the silicon evaporation temperature $T_{\max}$ = $T_{\mathrm{evap},\;\mathrm{Si}}$ = 3538 K. At *H* = 1.77 J/cm${}^{2}$, the heating phase duration (from 2100 K up to the maximum temperature $T_{\max}$) is $t_{\mathrm{heating}}$ = 20 ns and the cooling phase duration (from the maximum temperature $T_{\max}$ down to 2100 K) is $t_{\mathrm{cooling}}$ = 36 ns. The melt width $2\Delta r_{\mathrm{melt}}$ is about 6 µm ($\Delta r_{\mathrm{melt}}$ = 3 µm). The calculated circulation velocity $v_{c}$ during the heating phase for an average melt depth $d_{\mathrm{melt},\;\mathrm{heating}}$ = 340 nm is $v_{c,\;\mathrm{heating}}$ = 12.6 m/s, and that during the cooling phase for an average melt depth $d_{\mathrm{melt},\;\mathrm{cooling}}$ = 600 nm is $v_{c,\;\mathrm{cooling}}$ = 22.3 m/s. The total circulated distance during both heating and cooling phases is $L_{\mathrm{circ}}$ = 1.06 µm, which is about 15.8% of the total circumference $L_{\mathrm{melt},\;\mathrm{total}}$ of half of the melted pool ($L_{\mathrm{melt},\;\mathrm{total}}$ = 6.7 µm). For *H* = 4.25 J/cm${}^{2}$, the heating phase duration (from 2100 K up to 3538 K) is $t_{\mathrm{heating}}$ = 12 ns, the evaporation phase duration ($T_{\max}$ = $T_{\mathrm{evap},\;\mathrm{Si}}$ = 3538 K) is $t_{\mathrm{evap}}$ = 46 ns, and the cooling phase duration (from 3538 K down to 2100 K) is $t_{\mathrm{cooling}}$ = 65 ns. The half melt widths during the three phases–heating, evaporation, and cooling–are $\Delta r_{\mathrm{melt},\;\mathrm{heating}}$ = 2.5 µm, $\Delta r_{\mathrm{melt},\;\mathrm{evap}}$ = 5.25 µm, and $\Delta r_{\mathrm{melt},\;\mathrm{cooling}}$ = 5.25 µm, respectively. The melt depths during the three phases are $d_{\mathrm{melt},\;\mathrm{heating}}$ = 250 nm, $d_{\mathrm{melt},\;\mathrm{evap}}$ = 663 nm, and $d_{\mathrm{melt},\;\mathrm{cooling}}$ = 413 nm, respectively. Using average temperatures during the heating and cooling phases, the circulation velocity $v_{c}$ during the heating, evaporation, and cooling phases are $v_{c,\;\mathrm{heating}}$ = 10.7 m/s, $v_{c,\;\mathrm{evap}}$ = 26.5 m/s, and $v_{c,\;\mathrm{cooling}}$ = 8.3 m/s, respectively. The result is a total melt circulation distance $L_{\mathrm{circ}}\approx$ 1.9 µm, which is about 16.1% of the total circumference $L_{\mathrm{melt},\;\mathrm{total}}$ of half of the melt pool ($L_{\mathrm{melt},\;\mathrm{total}}$ = 11.8 µm). The circulation action in both pulse energy densities, 1.77 J/cm${}^{2}$ and 4.25 J/cm${}^{2}$, justifies the higher pre-exponential factor $D_{0}$ used in the model of Lill et al. [13] and in the present model. The reason is that the same maximum pre-exponential factor $D_{0}$ ($D_{0}$ = 8 × 10${}^{-4}$ cm${}^{2}$/s) is used for the simulation of the whole *H* range, as the circulated portion $L_{\mathrm{circ}}$/$L_{\mathrm{melt},\;\mathrm{total}}$ for *H* = 1.77 J/cm${}^{2}$ and 4.25 J/cm${}^{2}$ is almost the same–about 16%.
